# Comparison of survival in adult antiretroviral treatment naïve patients treated in primary health care centers versus those treated in hospitals: *retrospective cohort study*; Oromia region, Ethiopia

**DOI:** 10.1186/s12913-016-1818-3

**Published:** 2016-10-18

**Authors:** Abebe Megerso, Sileshi Garoma

**Affiliations:** Adama Hospital Medical College, P.O. Box 3092, Adama, Ethiopia

**Keywords:** Antiretroviral therapy, Survival, Lost-to-follow up, Oromia, Ethiopia

## Abstract

**Background:**

Antiretroviral treatment (ART) service scaling up has been practiced in the Ethiopia since 2006. Regardless of increasing number of primary health care centers providing the service, the existing hospitals are still overcrowded with ART service seeking patients may be because of the common belief that treatment outcome is better for hospital patients than those treated at the primary health centers. However, documented evidence comparing the treatment outcome for the two categories of health facilities is scarce in the study setting. The purpose of the current study was to compare major treatment outcomes among new patients treated at the two health facility categories.

**Method:**

Retrospective cohort study was implemented using secondary data from medical records collected between October 2010 and January 2014 in the selected health facilities. All patients (1895) who started the treatment in the facilities during the period were included in the study. Univariate analyses were made using descriptive methods such as frequency distributions and measures of central tendency. Bivariate and multivariate analyses were made using Kaplan Meier and Cox regression models respectively to compare the mean survival time between the two facility categories. *P*-value less than 0.05 was considered as statistically significant.

**Results:**

A total of 1895 patient records were followed for 27,990 person-months. Risks of unwanted treatment outcomes (death and lose-to-follow-up) were the same for both categories of patients. The median survival probability was similar to the facility categories (*P*-value = 0.11). Baseline performance scale III/IV (AHR, 2.4; 95 % CI: 2.0, 3.0), baseline WHO clinical stages III/IV (AHR, 2.8; 95 % CI: 2.3, 3.4), and low adherence (<95 %) to ART drugs (AHR, 3.4; 95 % CI: 2.8, 5.2) were the independent predictors of the unwanted treatment outcomes.

**Conclusion:**

Antiretroviral treatment service delivery at primary health care facilities did not compromise the treatment outcomes among adult ART naïve patients. This implies that, ART services decentralization can result in acceptable treatment outcome in less developed settings. Therefore, treatment requiring patients should be encouraged to start the treatment in either of the health facilities as early as possible.

## Background

Human immunodeficiency virus (HIV) has continued to be an enormous challenge worldwide. Since its recognition, HIV has infected close to 70 million people, and more than 34 million have died due to acquired immunodeficiency syndrome (AIDS) [1]. Although there is slight decline in the burden of the disease, it is still causing challenges to the world [1, 2]. Globally, there were an estimated 36.9 million people living with HIV, 2.0 million new HIV infections and 1.2 million deaths due to the disease in 2014 [3]. In sub Saharan Africa alone, about 25.8 million people were living with HIV and the region accounted for 70 % of the new infections in the world in the year [4]. Antiretroviral treatment (ART) scale up is ongoing globally [3]; although 14.9 million (only 40 % of the patients in need of the treatment) were on ART of which 13.5 million were in low and middle income countries in 2014 [4]. Ethiopia is the second most populous in Africa [5] and one of the seriously affected countries in sub Saharan Africa [2, 6]. The estimated prevalence of HIV/AIDS in Ethiopia was 1.5 % with urban and rural estimation of 4.2 and 0.6 %, respectively in the year 2011 [4].

Indeed, the use of ART drugs (the drugs prescribed to HIV/AIDS patients based on defined criteria) dates back to about two and half decades in developed countries, while its use started in 2003 in Ethiopia [7]. In this country, free ART service was launched to be given in public hospitals in 2005 and in primary health care centers in 2006 as part of the service scaling-up [8, 9]. The criteria used to start patients on ART in Ethiopia include WHO clinical staging, TLC-count and CD_4_- count as used in combination or separately [6, 10].

Accordingly, in Ethiopia the number of patients ever started ART was 439,301, of which 317,443 were on the treatment since in 2013 [8]. The number of ART providing facilities is increasing, (reaching 318 in Oromia region and 913 nationally) of which about 80 % are in primary health care centers [8, 11].

Comparing the survival of patients treated as relatively established health facilities and those treated in newly incoming primary health care centers can inform both the practitioners and the program mangers as to what is happening as ART service scaling up to mid-level health workers. Nonetheless, evidence showing what was happening to the survival of the adult patients who were enrolled into the treatment in hospitals and health centers was scarce. Therefore, this study compared the survival of adult patients who were new to ART between a hospital and health centers.

## Methods

### Study design, setting and period

A retrospective cohort design was used to analyze data obtained from medical records of patients on ART in southern central Oromia regional state. In Ethiopia, the health service delivery system has three tiers. The first tier is known as the primary health care unit where non physician clinicians and other less trained health workers provide basic and primary health care services. This tier includes health service delivery facility known as health center. The other two tiers are general hospitals and specialized referral hospital where more qualified practitioner provides medical services to the patients.

The two study settings compared in the current study were primary health care centers (the exposure setting) and hospitals. In the primary health care centers middle and low level health workers such as nurses and health officers (graduates of Bachelor of Science degree in public health) are responsible for patient care. The centers are out of major towns and less equipped with health care facilities. They have newly received the responsibility to provide ART services as a result of the service decentralization. As a result of lesser expertise of the health care providers and lesser health care facility in the primary health care centers, patients treated at this set up were considered to be exposed group. In the second health care facility, the hospitals, medical doctors are responsible to provide ART services. Hospitals are located in urban settings and have better health care facilities as compared to the primary health care centers. We analyzed and compared the major ART services outcomes using medical records collected between October 01, 2010 and January 30, 2014.

### Population

Source population for the current study was all new adult HIV/AIDS patients, who have ever started on highly active antiretroviral treatment (HAART) regimen in the study area and other similar areas. All public health facilities which have been providing ART service since October, 2010 in the study area were selected for the study. Adult ART naïve patients (15 years or older), treated in the selected health facilities were included in the study. Those patients recorded as transferred out to other health facilities to follow the treatment elsewhere for the whole duration since the transfer out were excluded from the study.

### Analysis

A total of 1895 medical records (all that meet the inclusion criteria) obtained from the standardized ART registers, 1307 and 588 from hospital and health center respectively were analyzed. Using EPI Info computer software sample size calculation formulae, and considering 95 % confidence level, 80 % power, unexposed to exposed group ratio of two and relative risk of two, total sample size of 900 could be statistically sufficient. We increased the sample size to more than double to get more reliable result. Variables with missing data were separately analyzed and compared for the two categories of the health facilities. All data collectors and supervisors were trained on the tools for consistency and supported by close supervision during data collection. EPI-Info version 6 was used for data entry [12]. The entered data were cleaned through the phase by phase screening after exporting to the SPSS statistical computer software version 20 [13]. This was done, first by sorting variables with incomplete values. Values missed at entry were completed from the hard copy of the collected data. Then, we ran separate frequencies for each variable to further check and clean the data.

Descriptive statistical methods were used in univariate analysis to generate frequencies and measure of central tendencies. The hypothesis of no survival difference among ART naïve patients treated in both health facility categories was also tested. Bivariate and multivariate analyses were made using Kaplan Meier and Cox regression models respectively, to compare the survival rate among patients of the two categories. We fit Cox regression model including all variables reported in Table 3. Survival rate is explained by the mean survival time of patients before death or lost to follow up (LTFU). Chi-square test was also used to compare categorized outcomes. If a patient misses the treatment follow up appointment for over a month after the appointment date and death could not be ascertained, then the patient was categorized as LTFU. In this study, we fitted survival model considering two different scenarios. In the first scenario, we considered the event of death and LTFU together as event of interest; while in the second scenario, we considered only confirmed deaths as event of interest. *P*-value less than 0.05 was considered as statistically significant.

**Table 1:** Socio-demographic characteristics of study participants; October, 2010 to January, 2014

Variables	Frequency	Percentages (%)
Age (*n* = 1895)		
15–29 30–39 40–49 > = 50	662 766 313 154	34.9 40.4 16.5 8.1
Sex (*n* = 1895)		
Male Female	834 1061	44.0 56.0
Marital status (*n* = 1895)		
Unmarried Married Divorced Separated Widowed	283 1043 140 169 260	14.9 55.0 7.4 8.9 13.7
Religion (*n* = 1895)		
Muslim Orthodox Protestant Catholic Other	545 984 307 21 38	28.8 51.9 16.2 1.1 2.0
Residence (*n* = 1895)		
Urban Rural	1376 519	72.6 27.4
Educational status (*n* = 1895)		
Illiterate Read and write Elementary school High school Diploma and above	545 90 724 474 62	28.8 4.7 38.2 25.0 3.3
Working situation (*n* = 1893)		
Employed by other Self-employee Jobless Student Other	246 701 910 14 22	13.0 37.0 48.1 0.7 1.2

**Table 2:** Comparison of mean survival of patients treated in Hospital versus that of Health center; October, 2010 to January, 2014

Facility	Mean survival (95 % CI)	*X* ^*2*^_*(1)*_	*P*-Value
Health center	31.3 (30.0,32.6)	2.6	0.11
Hospital	30.3 (29.4,31.3)

**Table 3:** Association between baseline variables and hazards of failure (death/LTFU) among ART patients in studied health facilities; October, 2010 to January, 2014

Variables	Crude	^b^Adjusted
	HR (95 % CI)	HR (95 % CI)
Baseline functional status		
Bed ridden/Sick ambulatory	2.5 (2.0,3.0)	2.4 (2.0,3.0)
Working functional status	Reference category	
Baseline WHO stage		
WHO stage III/IV	1.6 (1.3,2.1)	1.3 (1.0,1.6)
WHO stage I/II	Reference category	
Baseline CD_4_ count		
CD4 less than or equal 200cell/ml	1.1 (0.9,1.4)	1.0 (0.8,1.3)
CD4 less than 200cells/ml	Reference category	
Disease stage at ART start time^a^		
Advanced	3.3 (2.7,4.0)	2.8 (2.3,3.4)
Not advanced	Reference category	
CPT regular refilling		
No regular refilling	2.3 (1.0,5.2)	2.0 (0.9,4.5)
Had regular refilling	Reference category	
Adherence to ART		
Poor/Fair (<95 %)	3.6 (2.9,4.4)	3.4 (2.8,5.2)
Good (> = 95 %)	Reference category	
Facility category		
Health Center	0.8 (0.7–1.0)	0.9 (0.7–1.1)
Hospital	Reference category	

^a^Advanced disease stage is for patients in the WHO stage III/IV with CD_4_ cell count less than 200 cells/ml

^b^Adjusted for socio-demographic variables and all variables in the table

## Results

The medical records of 1895 ART naïve patients were analyzed. The ratio of hospital patients to that of health centers was about two to one. More than half of the patients, 1061 (56 %) were females and the median age was 24 years with an inter quartile range (IQR) of 15.8 to 31.80 years. Most of the patients, 1376 (70 %) were urban dwellers and over half of them, 1042 (55 %) were people within marital union. Only 531 (28 %) of the patients have attended high school level education or above. Half of the patients, 947 (50 %) were either self-employed or employed by others. That means, about 50 % of the study participants had certain monitory income from the work they were engaged in. House wives and other participants who were not involved in jobs with direct financial gains are considered jobless in this study (Table 1).

Data completeness rate was high (over 90 %) for all required variables in the study. From the total study participants, 1844 (97 %) had baseline body weight documented and the median weight was 50 kg with IQR 44 to 56 kg. Number of medical records with missing variables was compared for the two study settings to check for significant difference, and there was no difference in the missing data between the two categories of the health facilities. We compared the baseline data of the patients treated in the two facility categories such as CD_4_ count, body weight and WHO clinical stages. There was no difference in the baseline weight between hospital and health center patients (*P*-value = 0.35) and CD4 count (*P*-value = 0.31). The total of 1204 patients had advanced disease stage (WHO clinical state III/IV); of which 811 (67.4 %) were seen at hospital level.

The incidence rate of death in health center patients was 5.7 per 1000 person-months, while that of hospital patients was 6.3 per 1000 person-month of observation. The incidence rate of (LTFU) was 7.9 and 8.6 per 1000 person-months for health centers and hospital patients, respectively. Using two-by-two table with chi-square test statistics, we found that there was no statistically significant difference in incidence of both death and LTFU among patients treated at the hospital and health centers (*P*-values of 0.86 and 0.89) respectively. Among the health center patients with known performance scale, 511 (92.1 %) were improved from bed ridden or sick ambulatory to working performance scale; while 992 (76.1 %) of such hospital patients showed similar improvement. Immunological improvement (gain in CD_4_ cells count) was higher among health center patients than among hospital patients (*P*-value <0.001).

Considering the worst scenario (death or LTFU as an event of interest), the Kaplan Meier model was fitted to test for the difference between the mean survivals of patients treated in the two categories of the health facilities. The mean survival time was 31.3 months; (95 % CI: 30.0, 32.6) and 30.3 %; (95 % CI: 29.4, 31.1) months for health center and hospital patients respectively. The mean survival time was similar for patients of both health facility categories (*P*-value = 0.11) (Table 2).

**Fig. 1:**
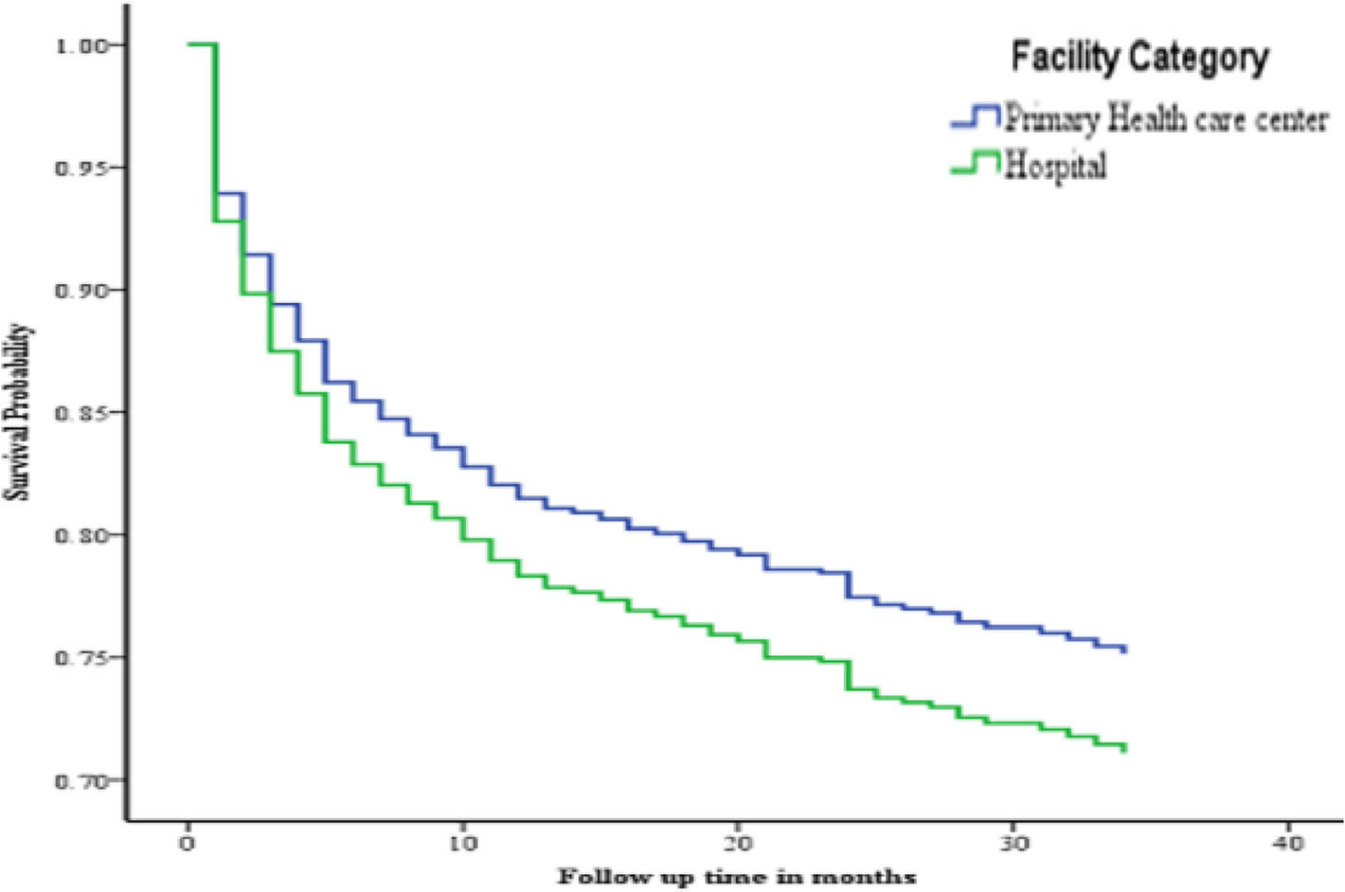
Survival Probability curve comparing primary health care centre ART patients with those of hospital based on risk of failure (Death and LTFU), October, 2010 to January, 2014

**Fig. 2:**
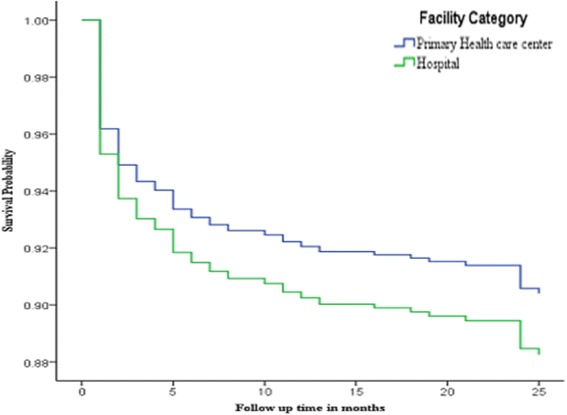
Survival probability curve comparing Health center and Hospital ART patients based on risk of death; October, 2010 to January, 2014

The survival probability curve was fitted for two extreme scenarios, the worst where LTFU cases were categorized with death as an event of interest and the other scenarios where only death was considered as an event of interest separately (Figs. 1 and 2). In both cases, although the difference was not statistically significant as shown in Table 2 above, patients treated at health center had better survival probability at any time during the follow up period.

There was no statistically significant association between the type of health facility attended by patients and risk of death and LTFU (*P*-value = 0. 11). The risk of death or LTFU was more than two times higher among patients who were bed ridden or sick ambulatory at admission compared to those who had working functional status with adjusted hazard ratio ((AHR), 2.4; 95 % CI: 2.0, 3.0). The risk was over three times higher among patients with advanced disease stage at the start of the treatment as compared to those who had no advanced disease (AHR, 2.8; 95 % CI: 2.3, 3.4). Risk of the event of interest (death and LTFU) was higher among male patients (AHR, 1.4; 95 % CI: 1.1, 1.7). There was no statistically significant association between the type of health facility attended by patients and risk of death or LTFU (*P*-value = 0. 11). Any adjusted hazard ratio reported for a particular variable in Table 3 is adjusted for all other variables in the table.

## Discussion

The current study showed the persistence of disproportionately high caseload to hospitals, with 1307 (69 %) were being treated at hospital. This high case load in hospital was similar to that of the 2009 Ethiopian national cohort analysis, which reported that 87.8 % of ART patients were receiving the treatment from hospitals [14]. The change of the hospital load from 87.8 to 69 % can be explained by the fact that ART service scaling up to health centers is ever increasing in the country [8, 15, 16]. Similar to the report of the national study done in Ethiopia [14], about two third of the patients (1428) were in the age range between 15 and 39 years. This higher proportion of the younger age may be due to higher disease burden in this age group [2, 4]. Moreover, the higher proportion of females patients 1061 (56 %) and urban residents 1376 (70 %) in the current study matches with an HIV prevalence trend in the country [4, 16, 17].

In the current study, the median baseline CD_4_ count was 130 Cells/ml and 140 Cells/ml for health centers and hospital patients, respectively. These counts were higher than the finding of other studies conducted in developing countries [18, 19]. This higher CD_4_ count at the initiation of the treatment could be attributed to the increasing access to the ART service which is facilitating for the early initiation of the treatment. We also confirmed that there was no difference between end-line mean counts of CD_4_ cells for the patients treated in either facility; and there was no difference in the treatment outcome for the facilities. The survival probability for patients of both facilities was similarly higher than 80 % at the fourth month of follow up.

In this study, we also found that being treated at the health center did not increase risk of death or LTFU to adult ART naïve patients. This finding was consistent with similar studies in Africa [18, 20–22]. The current finding was also similar to the recent Ethiopian national study finding which showed relatively better survival rate of patients treated in the primary health care centers as compared to those of hospitals (82 and 72 %, respectively) [23].^.^ This result was also in agreement with the findings of another study in South Africa and Cameroon which reported task shifting for HIV/AIDS care to relatively lower levels of the health system that did not compromise quality and rather associated with good ART outcomes [18, 20, 21, 24, 25].

Similarity, between the treatment outcomes in the two health facility categories could also be due strengthened mentoring of the service provision at both levels by trained health workers from outside the facilities. [26]. Strong predictors of death and LTFU were poor baseline functional status (being bed ridden or sick ambulatory), advanced disease stage and ART adherence less than 95 %. This findings were similar to study results from Brazil, and elsewhere [24, 27, 28] which showed that poor performance scale and WHO stage III/IV [6] as strong predictors of adult ART patients survival. Initiation and adherence to CPT was associated with event of interest and this result is in agreement with the result of the other study done in Ethiopia [19].

Limitation of the study was the fact that it was facility based where ascertaining the registered outcomes was impossible except relying on the ascertainments done by case managers working in respective health facilities. That means, registered outcomes such as death and LTFU were taken from health facility records without further ascertainment by the research team. But both categories of the health facilities have ascertainment mechanisms of the outcomes. The other limitation was the scope of the study which was confined to sub region, yet the results can be used in other parts of the region as there were similar ART service implementation strategies were followed in the whole region.

## Conclusion

In conclusion, the antiretroviral treatment outcome among ART naïve adult patients was not significantly different among patients treated at the primary health care centers and from those treated at hospital. The most important predictors of death and LTFU were having poor base line performance scale, advanced disease stage at the start of the treatment and poor adherence to ART regimens. Therefore, concerted efforts should be made by concerned bodies to encourage the patients to start the treatment in either of the health facilities as early as possible.

## Abbreviations

AHR: Adjusted hazard ratio
ART: Antiretroviral treatment
CI: Confidence interval
CPT: Cotrimoxazole prophylactic therapy
HAART: Highly active antiretroviral treatment
HR: Hazard ratio
LTFU: Lost to follow up
TLC: Total lymphocyte count
WHO: World Health Organization

## References

[1] World Health Organization: Fact sheet on HIV/AIDS, No 36. Accessed 25 July 2015 from http://www.who.int/mediacentre/factsheets/fs360/en/.

[2] Joint United Nations Programme on HIV/AIDS (UNAIDS): Report on the global AIDS epidemic. Accessed 10 July 2015 from http://www.unaids.org/sites/default/…/UNAIDS_Global_Report_2013_en_1.pdf.

[3] World Health Organization: Global update on HIV treatment 2013: results, impact and opportunities. Accessed 10 July 2015 from http://www.who.int/iris/bitstream/10665/85326/1/9789241505734_eng.pdf.

[4] Ethiopian central Statistical Agency: Ethiopia Demographic and Health Survey. 2011. https://dhsprogram.com/pubs/pdf/FR255/FR255.pdf.

[5] Central statistical Agency of Ethiopia: Population and Housing Sensus of Ethiopia; 2007. Accessed 23 July 2015 from http://www.csa.gov.et/newcsaweb/images/documents/pdf_files/…/Oromya1.pdf.

[6] Federal Ministry of Health-Ethiopia: New Integrated Guideline for Implementation of Antiretroviral Therapy in Ethiopia adopted from WHO. 2013. Accessed 22 July 2015. From http://www.ilo.org/wcmsp5/groups/public/---…/wcms_125385.pdf.

[7] Federal Ministry of Health, HIV/AIDS Prevention and Control Office (EHAPCO). Guidelines for Implementation of Antiretroviral therapy programme in Ethiopia. 2007. Accessed from http://www.ilo.org/wcmsp5/groups/public/---…/wcms_125385.pdf.

[8] FDR of Ethiopia Office of HIV/AIDS prevention and control (FHAPCO): Country progress report of response to HIV/AIDS. 2014. Accessed 13 July 2015. http://www.unaids.org/sites/default/…/country/…/ETH_narrative_report_2014….

[9] Wim VD, Katharina K, Guy K (2008). Scaling-up antiretroviral treatment in Southern African countries with human resource shortage: How will health systems adapt?. Soc Sci Med.

[10] World Health Organization: Consolidated Guidelines on the use of antiretroviral drugs for treating and preventing of HIV infection: Recommendations for a public health approaches. Available at www.who.int/hiv/pub/guidelines/arv2013/en/.

[11] Oromia Regional state Health Bureau: Annual performance report on HIV/AIDS prevention and control. 2014. Retrieved on July 12, 2015 from http://www.moh.gov.et/oromiahb.

[12] Dean AG, Dean JA, Coulombier D, et al.: Epi Info, Version 6: a word processing, database, and statistics program for public health on IBM compatible microcomputers. Centers for Disease Control and Prevention, Atlanta, Georgia, U.S.A. 1996.

[13] SPSS Inc: Statistical products and service solutions; 233 South Wacker Drive, 11th Floor Chicago, IL 60606–6412. 2007.

[14] Massaquoi M, Zachariah R, Manzi M (2009). Patient retention and attrition on antiretroviral treatment at district level in rural Malawi. Trans R Soc Trop Med Hyg.

[15] Federal Democratic Republic of Ethiopia HIV/AIDS Prevention and Control Office (EHAPCO). Progress towards implementation of the UN Declaration of Commitment on HIV/AIDS. 2014. Available from www.unaids.org/sites/default/files/country/.../ETH_narrative_report_2014.pdf.

[16] Assefa Y, Alebachew A, Lera M, Lynen L, Wouters E, Damme WV: Scaling up antiretroviral treatment and improving patient retention in care: lessons from Ethiopia. Globalization and Health 2014, 10 (43). Available from www.ncbi.nlm.nih.gov › NCBI › Literature › PubMed Central (PMC). 10.1186/1744-8603-10-43 PMC4046386 24886686

[17] Berhane Y, Mekonnen Y, Seyoum E, Gelmon L, Wilson D: HIV / AIDS in Ethiopia—An Epidemiological Synthesis. 2008. Accessed 11 Sept 2015 from http://www.siteresources.worldbank.org/INTHIVAIDS/…/EthiopiaSynthesisFinal.pdf.

[18] Fatti G, Grimwood A, Bock P. Better Antiretroviral Therapy Outcomes at Primary Healthcare Facilities: An Evaluation of Three Tiers of ART Services in Four South African Provinces. PLoS ONE. 2010;5(9). Accessed from journals.plos.org/plosone/article?id=10.1371/journal.pone.0012888. 10.1371/journal.pone.0012888 PMC2943483 20877631

[19] Alemu A, Sebastian MS. Determinants of survival in adult HIV patients on antiretroviral therapy in Oromiya, Ethiopia. Glob Health Action. 2010;3:(5398). Accessed from www.ncbi.nlm.nih.gov/pubmed/21042435. 10.3402/gha.v3i0.5398 PMC2967337 21042435

[20] Boyer S, Eboko F, Camara M, Abe C, Nguini MEO, Koulla-Shirog S (2010). Scaling up access to antiretroviral treatment for HIV infection: the impact of decentralization of healthcare delivery in Cameroon. AIDS.

[21] Zachariaha R, Ford N, Philips M, Lynch S, Massaquoi M, Janssens V (2009). Task shifting in HIV/AIDS: opportunities, challenges and proposed actions for sub-Saharan Africa. R Soc Trop Med Hyg.

[22] Assefa Y, Jerene D, Lulseged S, Ooms G, Damme WV. Rapid Scale - Up of Antiretroviral Treatment in Ethiopia: Successes and System - Wide Effects. PLoS Med. 2009;6(4). Accessed from www.ncbi.nlm.nih.gov › NCBI › Literature › PubMed Central (PMC). 10.1371/journal.pmed.1000056 PMC2667265 19399154

[23] Federal Ministry of Health-Ethiopia, HIV/AIDS prevention and Control office (EHAPCO). ART scale-up in Ethiopia; Success and challenges. 2009. Available from www.ncbi.nlm.nih.gov › NCBI › Literature › PubMed Central (PMC).

[24] Wandeler G, Keiser O, Pfeiffer K (2012). Outcomes of Antiretroviral Treatment Programs in Rural Southern Africa. J Acquir Immune Defic Syndr.

[25] Emdin CA, Millson P (2012). A systematic review evaluating the impact of task shifting on access to antiretroviral therapy in sub-Saharan Africa. Afr Health Sci.

[26] Federal Ministry of Health_Ethiopia: Health Sector Development Programme IV, Annual Performance Report. 2013. Accessed 9 July 2015 from http://www.moh.gov.et/…/Annual…Report…/4f5a6b33-3ef1-4430-a0a0-21fa1.

[27] Bhatta L, Klouman E, Deuba K, et al. Survival on antiretroviral treatment among adult HIV-infected patients in Nepal: a retrospective cohort study in far-western Region, 2006–2011. BMC Infect Dis. 2013;13(604). Available from bmcinfectdis.biomedcentral.com/articles/10.1186/1471-2334-13-604. 10.1186/1471-2334-13-604 PMC3880177 24369908

[28] Kebebew K, Wencheko E (2012). Survival analysis of HIV-infected patients under antiretroviral treatment at the Armed Forces General Teaching Hospital, Addis Ababa, Ethiopia. Ethiop J Health Dev.

